# Molecular Dynamics Simulations of the Short-Chain Fluorocarbon Surfactant PFH_X_A and the Anionic Surfactant SDS at the Air/Water Interface

**DOI:** 10.3390/molecules29071606

**Published:** 2024-04-03

**Authors:** Jinqing Jiao, Tao Li, Guangwen Zhang, Jing Xiong, Xuqing Lang, Xiaolong Quan, Yiwei Cheng, Yuechang Wei

**Affiliations:** 1 State Key Laboratory of Chemical Safety, SINOPEC Research Institute of Safety Engineering Co., Ltd., Qingdao 266071, China; zhanggw.qday@sinopec.com (G.Z.); langxq.qday@sinopec.com (X.L.); chengyw.qday@sinopec.com (Y.C.); 2 State Key Laboratory of Heavy Oil Processing, China University of Petroleum (Beijing), Beijing 102249, China; litaoreat@163.com (T.L.); xiongjing@cup.edu.cn (J.X.); qxl15993571310@163.com (X.Q.)

**Keywords:** short-chain fluorocarbon surfactants, hydrocarbon surfactants, synergy effect, molecular dynamics simulation, gas–liquid interface

## Abstract

The research and development of alternatives to long-chain fluorocarbon surfactants are desperately needed because they are extremely toxic, difficult to break down, seriously harm the environment, and limit the use of conventional aqueous film-forming foam fire extinguishing agents. In this study, mixed surfactant systems containing the short-chain fluorocarbon surfactant perfluorohexanoic acid (PFH_X_A) and the hydrocarbon surfactant sodium dodecyl sulfate (SDS) were investigated by molecular dynamics simulation to investigate the microscopic properties at the air/water interface at different molar ratios. Some representative parameters, such as surface tension, degree of order, density distribution, radial distribution function, number of hydrogen bonds, and solvent-accessible surface area, were calculated. Molecular dynamics simulations show that compared with a single type of surfactant, mixtures of surfactants provide superior performance in improving the interfacial properties of the gas–liquid interface. A dense monolayer film is formed by the strong synergistic impact of the two surfactants. Compared to the pure SDS system, the addition of PFH_X_A caused SDS to be more vertically oriented at the air/water interface with a reduced tilt angle, and a more ordered structure of the mixed surfactants was observed. Hydrogen bonding between SDS headgroups and water molecules is enhanced with the increasing PFH_X_A. The surface activity is arranged in the following order: PFH_X_A/SDS = 1:1 > PFH_X_A/SDS = 3:1 > PFH_X_A/SDS = 1:3. These results indicate that a degree of synergistic relationship exists between PFH_X_A and SDS at the air/water interface.

## 1. Introduction

As society and living standards rise, all types of uncontrolled fire behavior increase in frequency and pose a major threat to the safety of people’s lives and property. The development of effective fire-extinguishing chemicals is especially crucial. Aqueous film-forming foam (AFFF) is a synthetic foam used in firefighting, the petroleum and petrochemical industry, and other applications. It is one of the most effective technologies for extinguishing flammable liquid fires [1]. Fluorocarbon surfactants, especially long-chain fluorocarbon surfactants (C_8_–C_10_), such as perfluorooctane sulfonate (PFOS), are the essential surface-active materials in fire-extinguishing agents. Fluorocarbon surfactants have high surface activity and thermal stability, which can quickly promote the foam mixture to diffuse and deposit a dense foam layer on liquid fuel surfaces, and then effectively separate the fuel from the air [2]. However, some studies have revealed that conventional long-chain fluorocarbon surfactants (C_n_F_2n+1_, n > 6) are a type of toxic organic pollutant that is hard to degrade; their environmental pollution is persistent and bioconcentrated, which is a serious threat to the ecological environment. Therefore, long-chain fluorocarbon surfactants, especially PFOS, have been prohibited by the United Nations Environment Programme. The examination of novel fluorocarbon surfactants with low environmental contamination, non-bioaccumulation, and biodegradation has taken precedence [3].

Reducing the carbon atoms in perfluorinated radicals from C_8_–C_10_ to C_4_–C_6_ can enhance the biodegradability and reduce the toxicity of long-chain fluorocarbon surfactants [4]. Perfluorinated short-chain surfactants are a new type of fluorocarbon surfactant, which are considered environmentally friendly surfactants due to their lower biotoxicity and higher biodegradability than conventional surfactants. Additionally, these fluorocarbon surfactants have excellent surface properties [5,6]. Practical studies have shown that pure surfactants or one surfactant alone are frequently insufficient, and combining surfactants yields better results than using them separately. At the same time, mixed surfactant solutions can provide a number of advantages over pure surfactant solutions, including increasing the solubility and stability of surfactants, improving the adsorption and dispersion of surfactants, and changing the arrangement of the molecular structure [7]. These advancements are largely credited to the synergistic effects brought about by the use of numerous surfactants, which are crucial in understanding phenomena such as surfactant-based foaming, emulsification, and solubilization.

In recent years, the molecular characteristics of surfactants at gas/water interfaces have been extensively studied using molecular dynamics (MD) simulation to determine the relationship between molecular structure and interfacial characteristics, and how they relate to one another [8,9,10,11,12,13,14]. Molecular dynamics simulation is a powerful method, and it not only has a big application potential in researching the function mechanism between reagents and interfaces but also enables us to study the dynamic process from a molecular perspective [15,16,17]. Recently, many academics have investigated the molecular perspective of surfactant molecule aggregation morphology at the interface to explain the microcosmic mechanism of surfactants. For example, Wang et al. [18] performed molecular dynamics simulations of the synergistic adsorption behavior of mixtures of dodecylamine (DDA) and alcohol at the air/water interface. The effects of alcohols with various alkyl chain lengths on surface activity and aggregation behavior were investigated. By calculating the density distribution and radial distribution, it was discovered that the DDA/alcohol mixtures formed a dense hydrophobic film at the air/water interface and had a stronger synergistic effect than pure DDA did at the same concentration. The surfactants in the mixture also tended to be close to one another at the air/water interface as the alkyl chain length of the alcohols increased, making the mixtures tightly aggregated and having better surface properties. Additionally, the nonionic fluorocarbon/hydrocarbon surfactants at the air/water interface were studied using MD simulations to understand their structure and adsorption behavior by Zhang et al. [19]. It was found that fluorocarbon surfactants are more structurally ordered than hydrocarbons at the air/water interface and that changing one or two CH_2_ molecules for one or two CF_2_ molecules has no effect on the interfacial structure. Wang et al. [20] studied the adsorption behavior of cationic dodecylamine (DDA)/anionic sodium oleate (NaOL) at the air/water interface at various molar ratios. The radial distribution function and density distribution were calculated. The findings reveal that the mixed surfactants have more extended conformations and smaller tilt angles in their carbon chains than pure NaOL and DDA, their mixtures can form compact monolayers at the air/water interface, and their mixtures have strong synergistic effects. However, there are few reports on molecular dynamics simulations of short-chain fluorocarbon surfactants at the gas–liquid interface.

In this study, the properties of perfluorohexanoic acid (PFH_X_A) and sodium dodecyl sulfate (SDS) surfactant on the gas/liquid surface were studied by molecular dynamics simulation. The surface tension, number of hydrogen bonds, degree of order, density distribution, radial distribution function, and solvent-accessible surface area of mixtures of PFH_X_A and SDS surfactant were calculated to elucidate the synergistic mechanism of PFH_X_A and SDS at the air–liquid interface. Moreover, the effects of different molar ratios of mixed surfactant on the gas/liquid interface structure were discussed. Based on the findings of this investigation, MD simulations would be helpful for understanding the kinetics and interfacial behavior of these compound surfactants and for providing some theoretical guidance for the further preparation of more efficient foam-extinguishing agents.

## 2. Results and Discussion

### 2.1. Aggregation Behavior of Pure Surfactant at the Air/Water Interface

**Figure 1:**
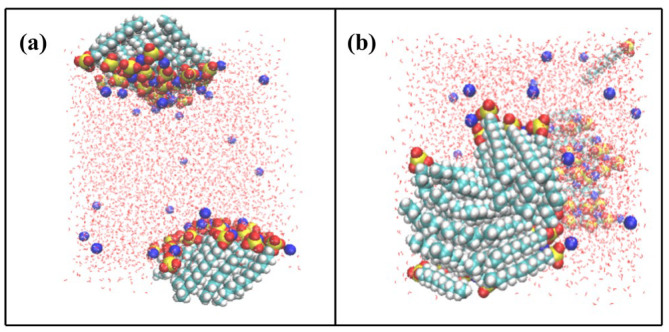
Snapshots of pure SDS surfactant at the end of gas–liquid interface simulations (the red, gray, blue, yellow and green spheres represent oxygen, hydrogen, sodium, sulfur and carbon atoms, respectively). (**a**) Side view of the system with SDS monolayers on the water surface and (**b**) top view of SDS monolayer.

**Figure 2:**
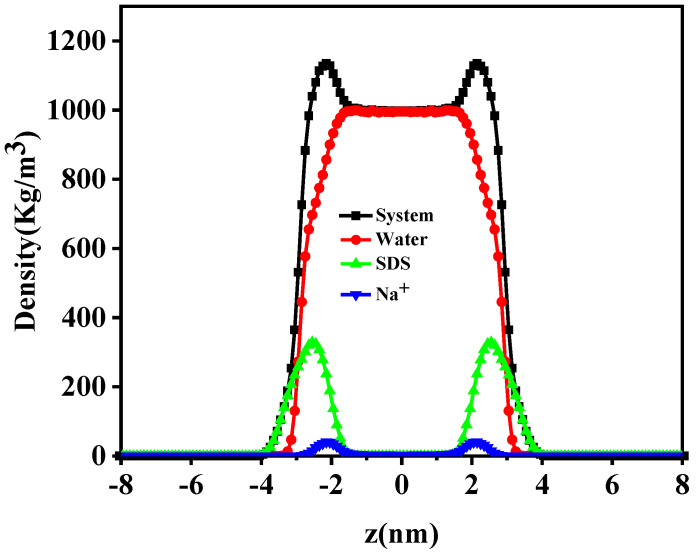
Density distribution profiles of pure SDS surfactant at the gas−liquid interface.

In this work, SDS surfactant is the major objective to be investigated. The snapshots of the final arrangement of SDS surfactant monolayers in the air/SDS/water/SDS/air system are displayed in Figure 1. The final arrangement of the pure PFH_X_A surfactant monolayers and density distribution are shown in Figures S2 and S3. Take SDS surfactants as an illustration, as shown in Figure 1. The hydrophobic carbon tail chains are extended towards the gas phase, the surfactant head groups are situated at the gas/water interface, and the carbon chains of the SDS surfactant are slanted at an angle towards the interface in comparison to the initial simulation. From the position of surfactant aggregation, it can be seen that the hydrophilic groups of surfactant all enter into the aqueous phase. In addition, a considerable portion of positively charged Na ions were found to be present in the interfacial region, close to the surfactant headgroup region, suggesting that Na ions bind to the surfactant headgroups due to electrostatic interactions. From the top view of Figure 1b, it is apparent that the SDS molecules aggregate at the air/water interface because of the strong hydrophobicity between the alkyl chains of the SDS surfactant. As shown in Figure 2, the density profiles along the *z*-axis of various components can be used to obtain comprehensive structural information. Because the two surfactant monolayers may operate separately and without impacting one another, the dual-interface model can mimic the aggregation behavior of the two-layer air/water interface. The distribution of the surfactant density profiles shows that the SDS hydrophilic head group enters the aqueous layer and undergoes hydration, whilst the alkyl chain is excluded from the aqueous layer and tends to the air. This phenomenon amply demonstrates that SDS has high surface activity and can adsorb at the air/water interface to produce a hydrophobic monolayer, thus reducing the surface tension of water. Notably, the water phase densities in the gas–liquid interfacial system are close to 0.998 g/cm^3^ for all systems, which is in line with the experimental density of 0.997 g/cm^3^ for water at ambient temperature. This demonstrates that the system model construction, force field, and parameter choices are plausible. Additionally, the findings of the molecular dynamics simulation are trustworthy and can be utilized to represent the surfactant aggregation behavior at the interface.

### 2.2. Aggregation Behavior of the Mixed System of PFH_X_A/SDS at the Air/Water Interface

**Figure 3:**
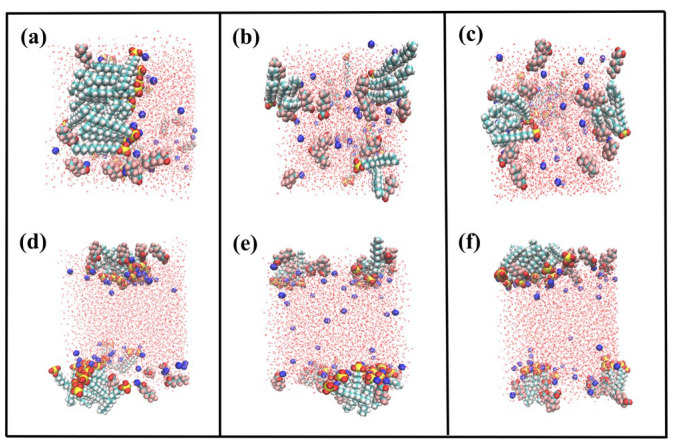
Snapshots of simulated boxes with different molar ratios of mixed surfactants (brown, red, gray, blue, yellow and green spheres represent fluorine, oxygen, hydrogen, sodium, sulfur and carbon atom, respectively). (**a**) PFH_X_A/SDS = 1:3; (**b**) PFH_X_A/SDS = 1:1; and (**c**) PFH_X_A/SDS = 3:1, shown in top view (**a**–**c**) and side view (**d**–**f**).

**Figure 4:**
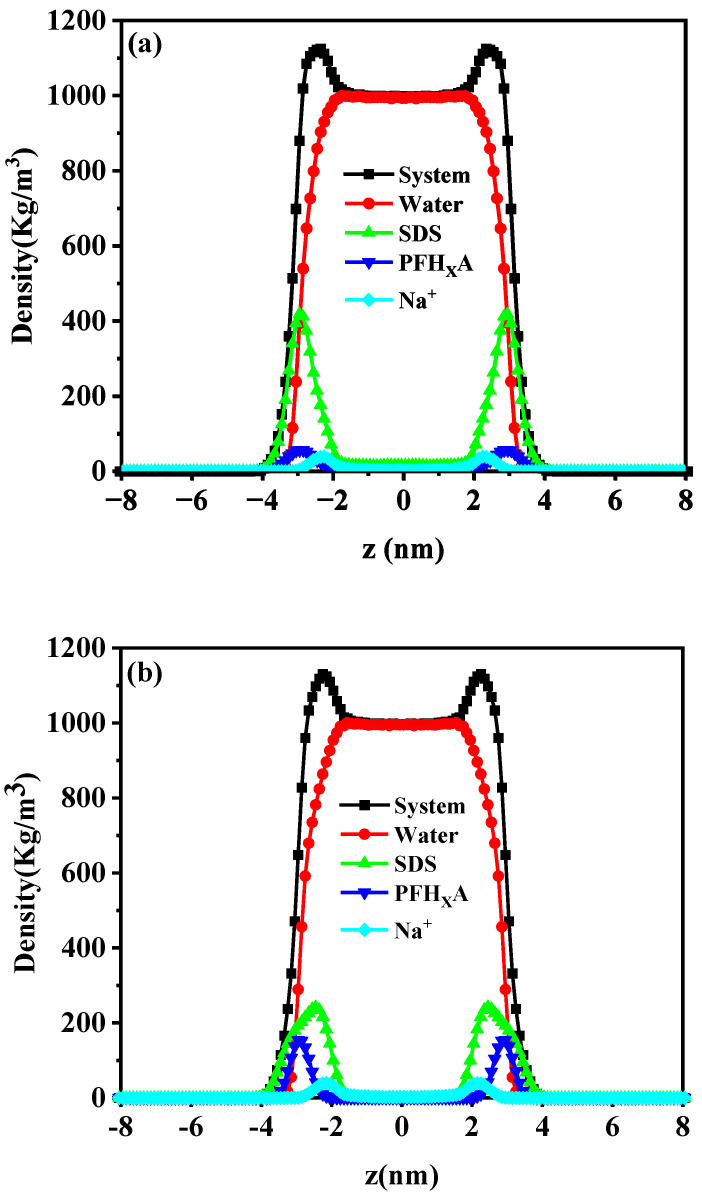

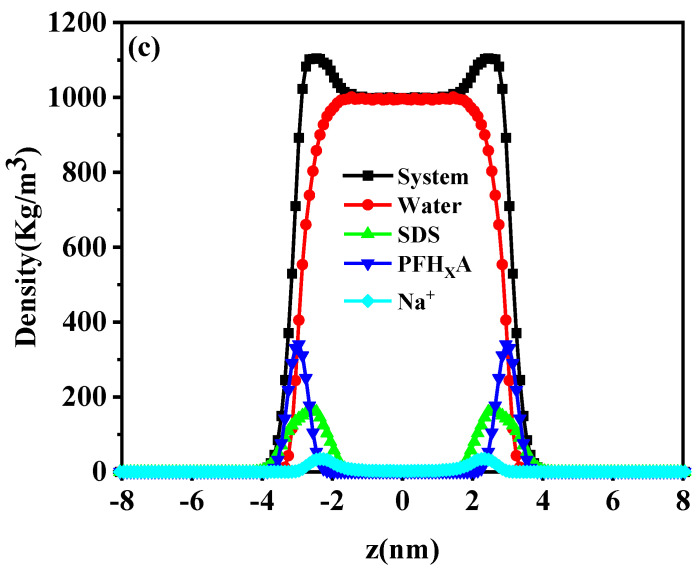
Density profiles of surfactants with different molar ratios along the *z*-axis direction. (**a**) PFH_X_A/SDS mixture ratio equal to 1:3, (**b**) PFH_X_A/SDS mixture ratio equal to 1:1, and (**c**) PFH_X_A/SDS mixture ratio equal to 3:1.

We calculated the density distribution of the mixed surfactant system to determine the composition of the interfacial layer and the distribution of its various components. A snapshot of the equilibrium of the monolayer of adsorbed PFH_X_A/SDS mixture is shown in Figure 3 and Figure S2. It can be seen that the aggregation behaviors of the PFH_X_A/SDS mixtures are different from those of pure SDS from the top and side views. The distribution of the mixtures at the air/water interface gradually becomes homogeneous with the increase of PFH_X_A and forms a dense adsorbed monolayer. As the mixed surfactant head groups hydrate and penetrate the aqueous phase, the carbon chains extend towards the air, and the nearby counterions are drawn to and move towards the interface, resulting in a corresponding decrease in the number of water molecules at the interface. According to the above-mentioned results, the hybrid PFH_X_A/SDS surfactant may possess sufficient surface activity in contrast to pure SDS surfactant. Additionally, the components’ density distributions along the *z*-axis in the mixed systems were also calculated. Figure 4 shows that PFH_X_A and SDS anionic surfactants exhibit a notable difference in peak distribution on the *x*-coordinate axis. PFH_X_A tends to be closer to the gas phase, while SDS is more likely to be in close contact with the aqueous phase. The surfactant mixture forms a stiff film at the air–water interface as a result of the hydrophobic interactions between the alkyl chains. Furthermore, more short-chain fluorocarbon surfactant molecules are found in the clefts of the hydrophobic chains of SDS, generating mixed adsorption, whereas a smaller number of PFH_X_A molecules compete with SDS for adsorption. Due to PFH_X_A’s hydrophobic nature, the monomolecular film of the surfactant became more homogeneous with increasing PFH_X_A ratio. The minimum peak distance between PFH_X_A and SDS is achieved when their mixing ratio is 1:1, suggesting that the two surfactants were well-compatible. The findings demonstrated that at the air/water interface, the PFH_X_A/SDS mixed surfactants displayed high surface activity. The interfacial density distribution cannot be used as an evaluation criterion to assess the interfacial performance, even though it may partially reflect the synergistic impact.

In order to confirm the equilibrium conditions of the system, the root mean square deviation (*RMSD*) of the SDS + PFH_X_A surfactant combination was then assessed on a simulated time scale [21]. In this study, *RMSD* is used to analyze the square root of the mean squared atomic deviation between the mixed surfactant initial conformation at time t = 0 and the conformation at time t [22,23]. Equation (1) determines this characteristic (reported in nm).

(1) $$RMSD=\sqrt{\frac{1}{N}\overset{n}{\underset{i=1}{\sum }}X_{i}^{2}}$$

where *N* stands for the overall number of molecules of ionic surfactant in the system. The *X_i_* term is the distance between the initial conformation of the identical ionic surfactant in the system and the conformation of the ionic surfactant at instant.

**Figure 5:**
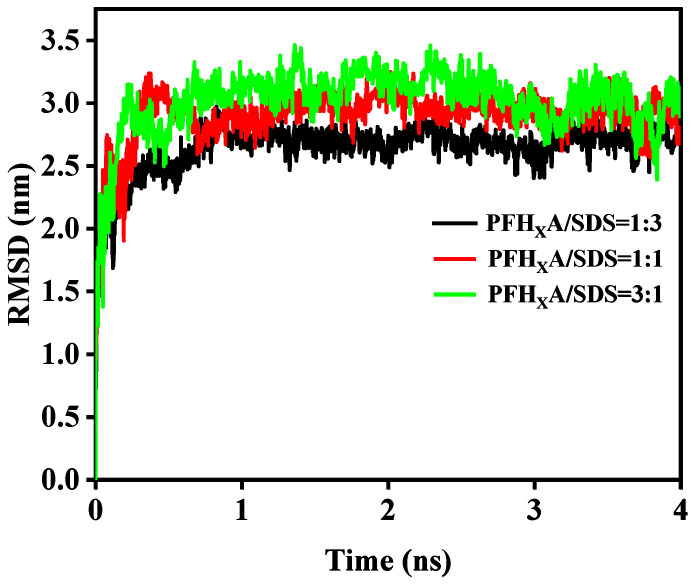
Average RMSD for the SDS surfactant in the mixed monolayers.

Figure 5 displays the root mean square deviation (*RMSD*) of SDS over time with mixed surfactants. The root mean square deviation (*RMSD*) of PFH_X_A is shown in Figure S4. The MD simulation starts with a significant rise in the *RMSD*. However, the *RMSD* curve changed less after 1 ns. The average *RMSD* values of SDS surfactant in the mixed system were (2.71 ± 0.12) nm, (2.95 ± 0.15) nm, and (3.21 ± 0.37) nm, respectively. These *RMSD* measurements show that the SDS surfactant in the mixed monolayer underwent a significant shift at time t in comparison to its starting state at time t = 0. Furthermore, the *RMSD* variation of the SDS surfactant becomes very minor after 1 ns, indicating that there has been no significant change in conformation and that the system has reached equilibrium by 1 ns into the simulation [24].

### 2.3. Surface Tension of the Mixed System of PFH_X_A/SDS

Surface tension is a crucial parameter for evaluating the interfacial characteristics of surfactants. In this study, the surface tension is computed by first calculating the pressure tensor in each plane, and then combining the box parameters and the energy file obtained from the simulation to calculate the surface tension. The surface tension is calculated using Equation (2):

(2) $$\gamma \;=\frac{1}{2}L_{Z}[P_{ZZ}-\frac{1}{2}(P_{XX}+P_{YY})]$$

where *L_Z_* is the dimensional length of box in the *Z* dimension, *P_XX_* and *P_YY_* represent the tangential pressure tensor components parallel to the interface, and *P_ZZ_* represents the component perpendicular to the interface. Equation (2) was used to determine the interfacial tension of water in this investigation, which was determined to be 69.62 mN/m. This result is consistent with the surface tension of water (71.8 mN/m) measured using an automatic surface tension meter (QBZY-3). It illustrates that the interfacial tension results obtained are reliable and that the force field and simulation settings utilized throughout the simulation process are suitable. As shown in Table 1, the interfacial tension value of the system is clearly lower after the addition of the surfactant, showing that the addition of surfactant molecules may successfully lower interfacial tension at the gas–liquid interface. The effectiveness of five different surfactants in reducing interfacial tension varies, with surfactant PFH_X_A being superior to SDS at the gas–liquid interface. In the complexed systems, the addition of PFH_X_A decreases the amount of hydrocarbon chains adsorbed on the surface and increases the amount of fluorocarbon chains adsorbed because it competes with SDS for adsorption. When the mixture ratio of PFH_X_A to SDS is 1:1, the adsorption capacity and close contact of hydrocarbon and fluorocarbon chains achieve optimal, and the lowest value of interfacial tension is reached. When the mixture of PFH_X_A continues to increase, the proportion of the adsorbed amount on the surface of PFH_X_A increases, and the surface tension increases instead, gradually approaching the surface properties of the single component of PFH_X_A.

### 2.4. Molecular Orientation of Hydrocarbon Chains of Ionic Surfactants

The ordering parameter in surfactant monolayers can count the molecular surface configuration at the ends of hydrophobic tail chains. The designated particles in the alkane chains are first selected as the start and end points, respectively, in order to establish the orientation vectors of the alkane chains in the surfactant molecules. The angle between the two points and the interface normal is then examined by connecting the two points. The ordering parameter can be obtained from Equation (3).


(3) $$S_{z}=\frac{1}{2}(3\;\cos^{2}\left(\theta \right)-1)$$

**Figure 6:**
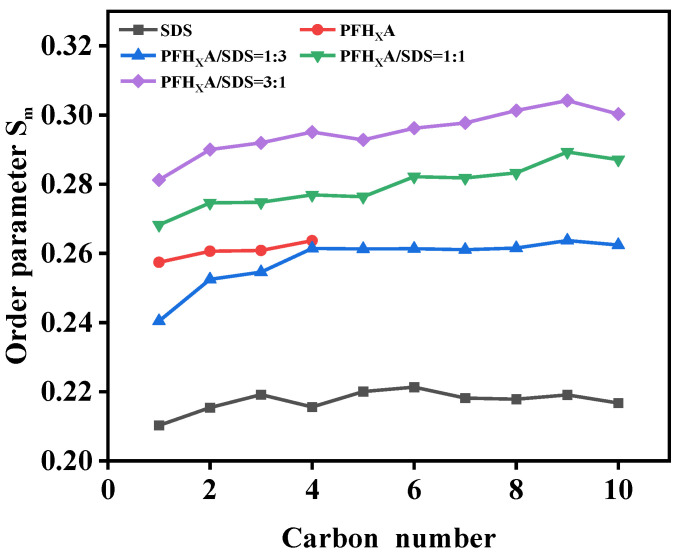
Ordering parameters at the gas–liquid interface with different systems.

In Equation (3), the vector between the carbon atom connected to the hydrophilic group (C_n_ atom) and the next carbon atom (C_n+1_ atom) forms an angle with the molecular axis of the hydrocarbon chains of ionic surfactants. The range of the computed order parameter should be −1/2 to 1. A value of −1/2 indicates an ordered arrangement of surfactant molecules along the perpendicular *z*-axis that is completely ordered in its direction, whereas a value of 0 for the order parameter indicates an isotropic arrangement [25,26,27]. As part of this investigation, an index file including all of the alkyl tail chain carbon atoms will be constructed in order to average the orderliness parameter over similar molecules in the system. The surfactant tends to be more perpendicular to the interfacial direction as the order parameter value increases because the direction of the line connecting the two atoms in the surfactant molecule becomes smaller in angle with the *z*-axis. It is not advantageous for surfactant tail chains to cover the water surface in this scenario. Instead, the angle between the direction of the atoms and the normal approaches 90° the lower the value of the order parameter, and the more it tends to be parallel to the interfacial direction. At the gas–liquid interface, we measured the ordering parameter of pure surfactant and PFH_X_A/SDS hybrid system. The findings are depicted in Figure 6.

The ordering parameters of all the systems can be shown to be positive in the figure, which illustrates that surfactants tend to adsorb and arrange themselves in a manner parallel to the *z*-axis at the air/water interface. By calculating the ordering of the hydrophobic chain segments of several surfactants at the gas–liquid interface, it was found that the ordering of the hydrophobic section in the surfactant compounding system was significantly greater than that of a single surfactant Compared to pure SDS surfactants, PFH_X_A has a higher degree of ordering because of its shorter carbon chain. The ordering of the interfacial arrangement for the poorly ordered SDS anionic surfactant system can be significantly changed by adding a specific amount of the short-chain fluorocarbon surfactant PFH_X_A, indicating the strong synergy between the two surfactants. The surface tension and the micelle concentration of the surfactant complex system are lower, which is more conducive to the development of mixed micelles or mixed adsorption layers. The degree of ordering progressively increases and subsequently declines in the pure SDS system, and in this case, the tails of the surfactants are often not parallel to each other. In complex surfactants, as the amount of PFH_X_A in the system grows, the interfacial layer gradually becomes more ordered, and the hydrocarbon chains of the surfactants are more perpendicularly oriented with respect to the interface (see Figure 3a through Figure 3c). This is mainly due to the insertion of PFH_X_A molecules between the surface-active ions of SDS when the two are compounded, resulting in a weakening of the electrical repulsion between the polar heads of the original SDS surfactant. At the same time, the hydrophobic interactions between the carbon chains of the two surfactant molecules promote a more compact and orderly arrangement of the surfactant molecules in the compounding system. When the mixture ratio of PFH_X_A to SDS was 3:1, the order parameter showed the greatest value, suggesting that the insertion of PFH_X_A molecules between active molecules on the SDS surface is saturated at this mixture ratio. Surfactant molecules generate the most ordered monolayer membrane structure at the interface. This result is in line with the explanation in the preceding section that the complexed surfactants in the air/water interfacial system have low interfacial tension.

### 2.5. Radial Distribution Function (RDF)

#### 2.5.1. Interaction of Surfactant Hydrophilic Groups with Water

**Figure 7:**
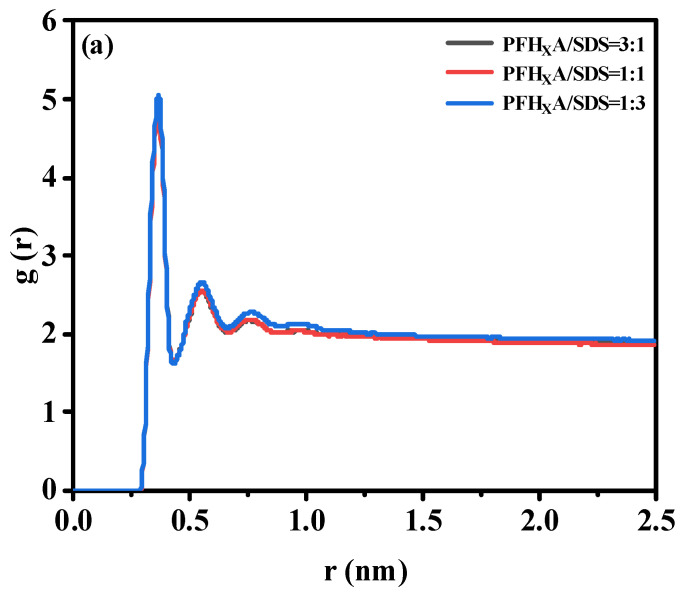

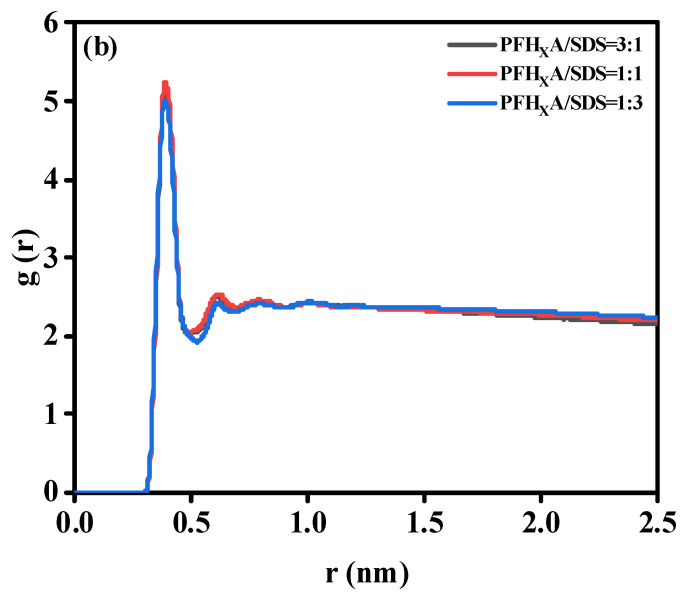
Radial distribution functions of (**a**) sulfur atoms and water in SDS headgroups and (**b**) carbon atoms and water in PFH_X_A headgroups in different systems.

The interfacial properties of surfactants are mostly determined by their interaction with water. Hydrophilic groups and water molecules engage in robust hydrogen bonding interactions. Therefore, in order to quantitatively characterize their interaction, the radial distribution function (RDF) between the head group and the water molecule was calculated, as shown in Figure 7. It is evident that the RDF forms of the three systems are similar. Therefore, adding PFH_X_A does not break the water shell surrounding SDS. It has been demonstrated that the interaction between the headgroup and the water molecule is largely determined by the first hydration water shell surrounding the headgroup [28], and in this paper, the first peak is chosen to study this interaction. In Figure 7a, the g(r) of the -OSO_3^−^_-OW pair shows that the system formed by the mixed PFH_X_A/SDS monolayer has four peaks at 3.56, 5.46, 7.64, and 9.58 Å (corresponding to the system without PFH_X_A surfactant), but the water molecules at a distance of 9.58 Å from the -OSO_3^−^_ group are considered to be unbound water. The peak at 3.56 (first peak) shows that there is a strong interaction between the oxygen atom of the -OSO_3^−^_ head group and the hydrogen atom of the water molecule, forming a hydrogen bond. Additionally, as the concentration of PFH_X_A in the air/water increased, the peak height of the first peak of the -OSO_3^−^_-OW pair decreased. As can be observed, the initial peak intensity decreases in the following order: PFH_X_A/SDS = 1:1 > PFH_X_A/SDS = 3:1 > PFH_X_A/SDS = 1:3. This trend might be explained by the addition of PFH_X_A, which significantly lowers the concentration of Na ions near -OSO_3^−^_. Na ions are poorly hydrated, and when they interact with -OSO_3^−^_ groups, they can release some water molecules. Water molecules are more likely to clump together with the -OSO_3^−^_ group as the quantity of Na ions in the area around it drops. It is demonstrated that COO- in the hydrophilic head group of PFH_X_A significantly affects the structure of water. Similar results were obtained from the RDF results of COO^−^ and water head groups, as shown in Figure 7b. Hydrogen bonding as well as nearby hydrogen bonding interactions between COO^−^ and water are represented by these two water shells around COO^−^. The RDF curve flattens down with increasing distance due to decreased hydrogen bonding interactions between COO^−^ and water. The difference in the intensity of the first peak compared to the second peak is significant. The decreasing order of the intensity of the first peak is: PFH_X_A/SDS = 1:1 > PFH_X_A/SDS = 3:1 > PFH_X_A/SDS = 1:3. The higher interfacial contact and lower interfacial tension are shown by the larger first peak intensity. Therefore, the surface activity should be weakened in the same order, and the PFH_X_A/SDS blend surfactant with a molar ratio of 1/1 showed good effectiveness in lowering surface tension.

#### 2.5.2. Interactions between Surfactants and Counterions

**Figure 8:**
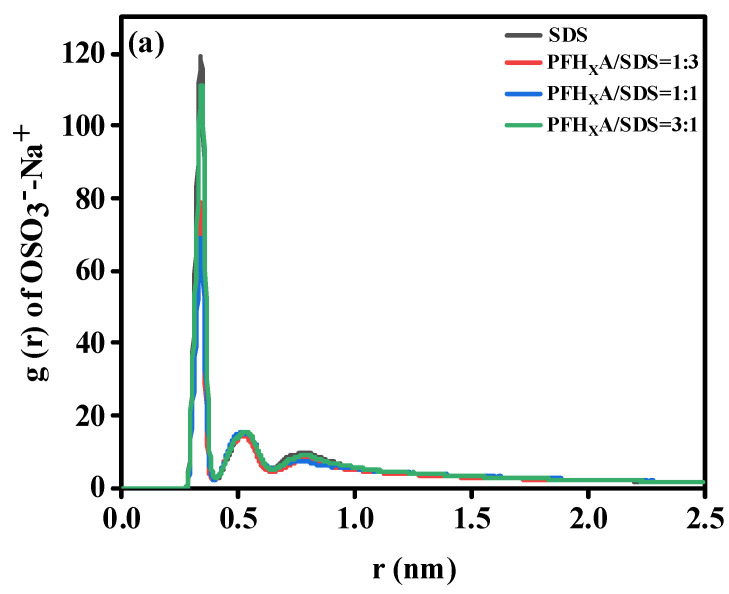

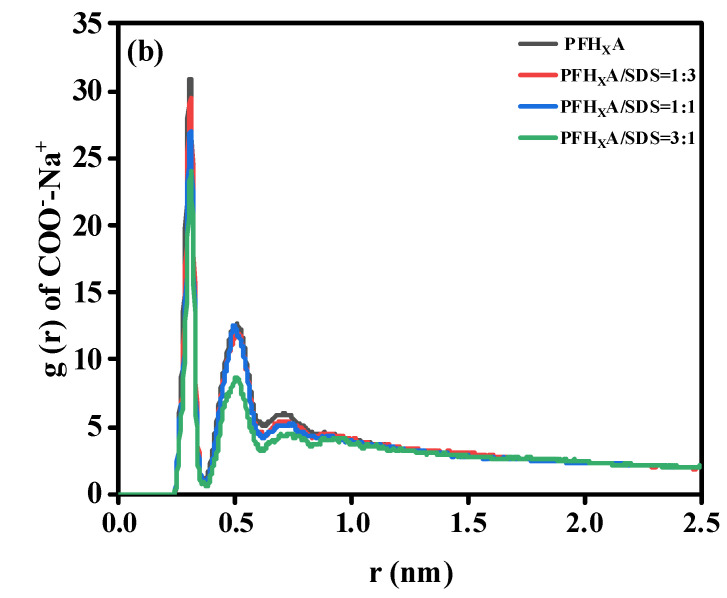
Radial distribution functions of (**a**) SDS headgroups and (**b**) PFH_X_A headgroups and their counterions in different systems.

Surfactant interfacial aggregation is impacted by counterions. The graphic displays the radial distribution function (RDF) of counterions for PFH_X_A and SDS headgroup pairs in various systems. The COO^−^ and -OSO_3^−^_ atoms in the PFH_X_A headgroup and the SDS headgroup, respectively, are used to indicate the locations of the headgroups. Figure 8a shows that there is just one significant peak, which is at around 3.38. Nearly every Na ion in pure SDS is found in the -OSO_3^−^_ group, which is positioned at a distance of around 3.4. As the fraction of PFH_X_A rises, the peak declines noticeably, indicating that the interaction between Na ions and the -OSO_3^−^_ group is less. This behavior is brought about by the potent competition adsorption between the SDS -OSO_3^−^_ group and the COO^−^ group in surfactant mixtures, which inhibits the -OSO_3^−^_ group from attaching to the Na ions. Figure 8b displays the RDF of the sodium ion and the PFH_X_A head group. The sodium ion and the carboxyl carbon atom exhibit two primary peaks; the first peak is around 3.93 and the second peak is approximately 5.89. The same result was obtained from the COO^−^ group interaction with the sodium ion, indicating that the addition of the -OSO_3^−^_ group reduces the COO^−^ group interactions with the sodium ion.

#### 2.5.3. Interactions between PFH_X_A and the Anionic Surfactants

**Figure 9:**
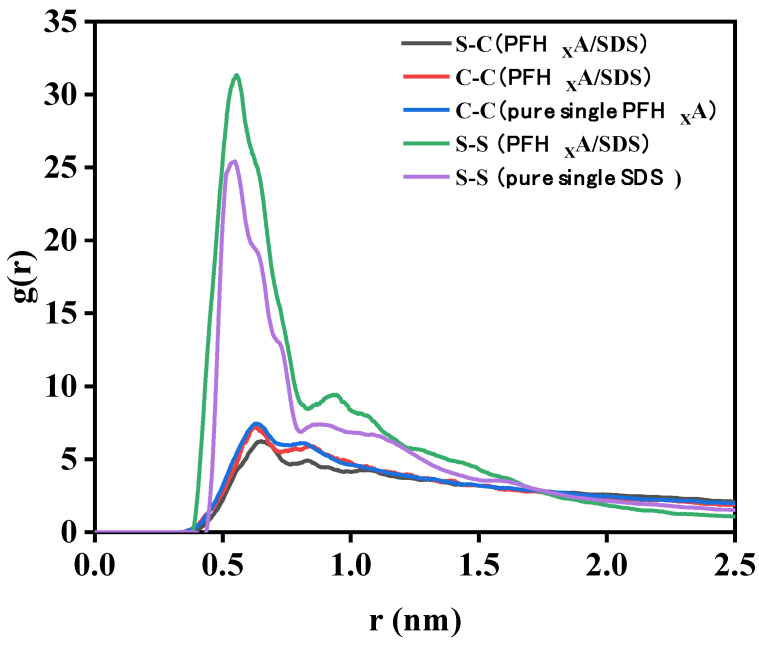
RDFs for the C atoms and S atoms in the PFH_X_A/SDS system.

At the interface between air/water, we investigated the interactions between the polar groups of a PFH_X_A/SDS combination at a ratio of 1:1. In PFH_X_A/SDS surfactant mixtures, the radial distribution function (RDF) of the carbon atoms in the carboxyl group and the sulfur atoms in the sulfonate group was computed, and the results are shown in Figure 9. The characteristic peak of the S-S atom RDF in the pure SDS system is approximately 3.82 Å, which is also the same characteristic peak as the mixed system. This suggests that the configuration of the sulfonic acid groups is unaffected by the addition of short-chain fluorocarbon surfactants. Similarly, the carbon atom RDF distribution remains unchanged when SDS is added to PFH_X_A.

### 2.6. Solvent Accessible Surface Area

**Figure 10:**
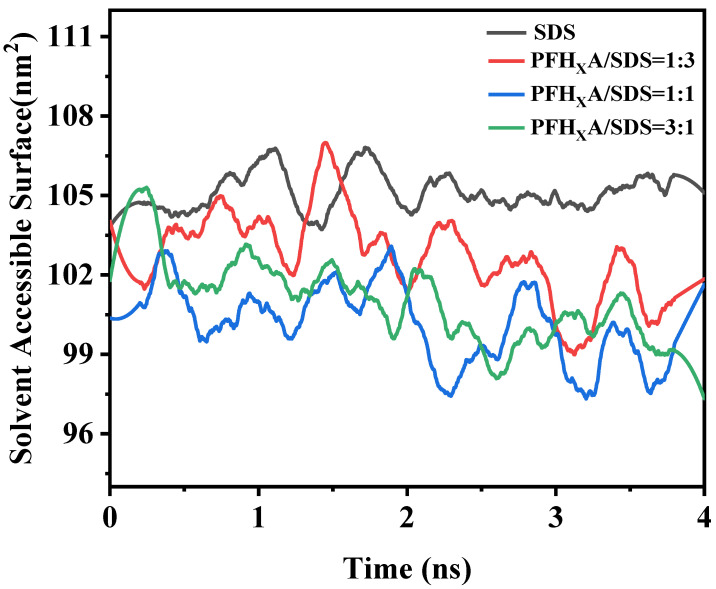
Variation of solvent accessible surface area (SASA) of surfactant molecules with simulation time.

The hydrophilic group occupied area is the region that the hydrophilic groups in the surfactants occupy; changes in this region can be intuitively interpreted to be changes in the surfactant aggregation pattern at the interface. Analyzing the soluble surface area of surfactants can reveal more information about the simulated system. This section used the g-sasa function to calculate the area occupied by the hydrophilic groups of the four surfactants, as shown in Figure 10. For the duration of the set simulation time, each curve of system keeps rising and falling. In pure SDS, the hydrophilic group occupied area is the largest. When PFH_X_A was added to the SDS surfactant, PFH_X_A and SDS competed for adsorption at the interface, and the hydrophilicity of the hydrocarbon chains of SDS caused them to be preferentially adsorbed at the gas–liquid interface. The quantity of fluorocarbon chain that was adsorbed at the interface increased as the molar fraction of PFH_X_A increased, while the amount of hydrocarbon chain that was adsorbed reduced. Meanwhile, and the fluorocarbon chain was arranged more tightly, the adsorption amount and the tightness of the arrangement of hydrocarbon chain and fluorocarbon chain reached the optimal level when PFH_X_A/SDS = 1:1. Continue to increase the molar fraction of PFH_X_A, when PFH_X_A/SDS = 3:1, the amount of PFH_X_A is absolutely dominant, the gas–liquid interfacial tension is elevated. It gradually converges to the nature of the one-component aqueous solution of PFH_X_A, and the surfactant SASA is elevated. This result further shows that a more solvent-accessible surface might be achieved by mixing surfactants in an appropriate ratio.

### 2.7. Number of Hydrogen Bonds

Hydrogen bonds are significant for analyzing the outcomes of molecular dynamics simulations. To supply a quantitative description of the hydrogen bond that exists between the water molecule and the polar head group of the surfactant. Apply the geometric criterion that establishes whether hydrogen bonding is present or absent: the H-O…H angle must be less than 30° and the distance between the chosen acceptor and donor must be less than 3.5 Å. With this definition, the quantity of hydrogen bonds that were created between the water molecules and the surfactant was computed [29].

**Figure 11:**
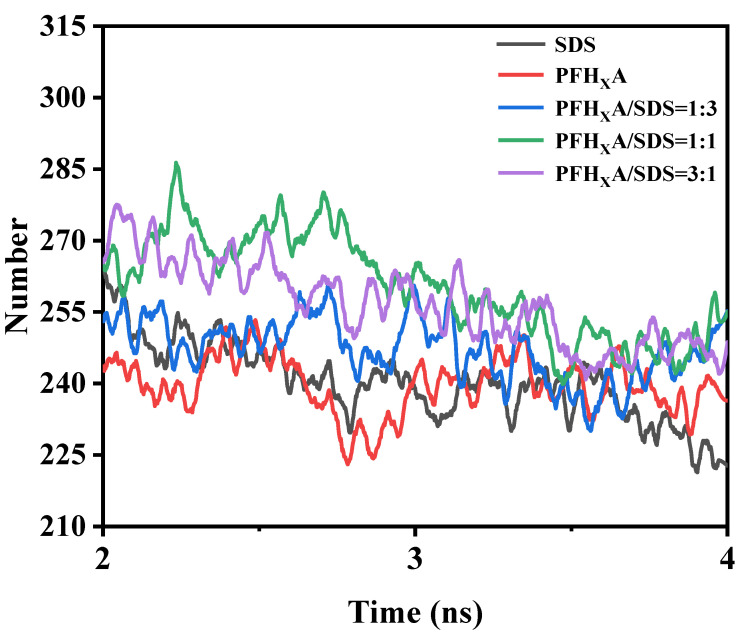
Variation with time in the number of hydrogen bonds between surfactant polar head groups and water molecules.

In each system, the quantity of hydrogen bonding is obviously different, as can be seen in Figure 11. In the pure surfactant system, the least amount of hydrogen bonds were generated between surfactant and water molecules. At a PFH_X_A/SDS mixing ratio of 1:1, the system hydrogen bonding capacity between surfactant and water molecules reached its maximum. According to this, PFH_X_A and SDS surfactant can have a significant synergistic effect when combined, as opposed to the pure surfactant system, which further verifies that PFH_X_A/SDS is a hybrid surfactant that can be used to reduce surface tension.

## 3. Simulation Method and Details

### 3.1. Modelling

**Figure 12:**
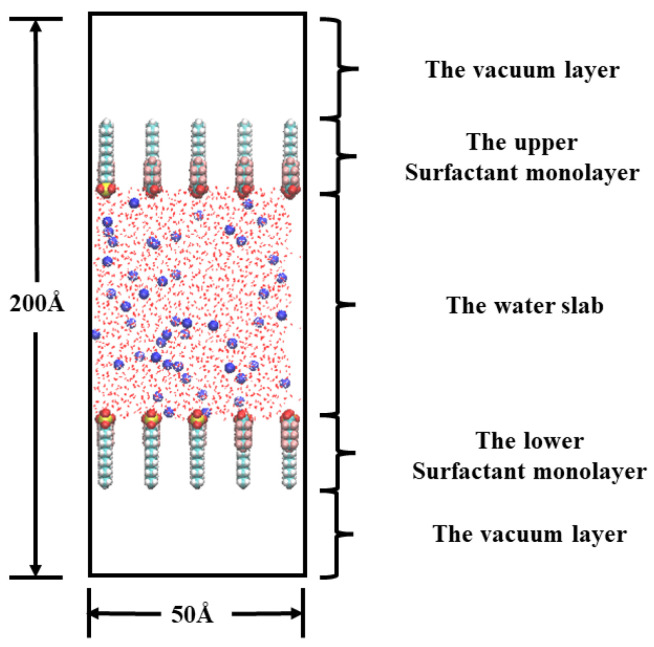
Initial configurations of mixed PFH_X_A/SDS surfactants in an air/water interfacial system.

MD simulations were conducted under the NVT ensemble using the anionic short-chain fluorocarbon surfactant perfluorohexanoic acid (PFH_X_A) and the hydrocarbon surfactant sodium dodecyl sulfate (SDS) as model surfactants [30]. The 2D and 3D molecular structures of PFH_X_A and SDS are shown in Figure S1. All the surfactants used in this paper were optimized by Gaussian 09 [31] software at the B3LYP/6-31G (d, p) level and the optimized structures are shown in Figure 12. The initial structure was constructed by PACKMOL software (version 20.2) as well as solvate and gmx editconf in GROMACS 2019 software for all conditions [32,33,34,35].

The sandwich model system should be built as seen in Figure 1. First, the spatial box dimensions were established. In the spatial right-angled coordinate system, the side lengths in the X, Y, and Z axes were set to 50 Å, 50 Å, and 200 Å, respectively. Water molecules were added in the center of the box, and the thickness of the water molecule layer was 50 Å, isolating the interaction between the upper and lower surfactant layers, and then two layers of the specified number of mixed surfactant molecules consisting of PFH_X_A and SDS were placed on the upper and lower sides of the aqueous solution. The hydrophobic chain segments of the surfactants are situated away from the water surface, while the hydrophilic groups are oriented toward the water molecules. The surfactant molecular chain is in a completely expanded conformation and is positioned vertically in the X–Y plane. The center of the box is also where the system center of mass is located. An air/surfactant/water/surfactant/air interfacial system is created by setting up a vacuum (gas phase) above and below the box. The vacuum length on the *z*-axis should be sufficient to completely minimize the system effect of periodicity. To maintain the system’s electrical neutrality, a certain number of sodium ions were added randomly to the water slab. Table 2 displays the total quantity of water molecules and surfactant molecules present in each of the simulated systems.

### 3.2. Simulation Details

All the simulations were performed with the GROMACS 2019 software package [36,37]. The CHARMM force field was employed in the simulations, and the SPC/E model was used to represent the water molecules model [38]. It accurately describes the dielectric and thermodynamic properties of water molecules. The Sobtop tool (version 1.0) [39] was used to create topology files, which were then manually modified to fit the simulated structures in the force field library. For all surfactant molecule structures, the all-atom model was applied. Multiwfn was used to create the atomic charge sums of the molecules of surfactants [40].

The equations of motion are solved by the Verlet algorithm, and the truncation radius of the VDW interaction is set to 12 Å for the calculation, which is set to be uncorrected in the long-range part of the van der Waals interaction. The PME (Particle Mesh Ewald) method is used to handle the long-range electrostatic interactions in this regime. To speed up the calculation, the system energy minimization (EM) is first switched from the steepest descent method to the conjugate gradient (CG) method. The maximum force is then set to 100 kJmol^−1^·nm^−1^, the number of energy minimization steps to 10,000, and the simulation step size to 2 fs, and the v-rescale thermostat was used to manage temperature [41]. The LINCS algorithm was used to restrict the bond lengths with a relaxation time constant of 0.1 ps [42]. Applications of periodic boundary conditions in all directions were made to the systems, and trajectories were recorded every 10 ps. Following initialization, the NVT system is then subjected to a 4 ns simulation since the conformation does not significantly change beyond that time, and the system potential energy stays stable. For statistical analysis, the findings of the final 2 ns were used to analyze molecular dynamics simulation. The GRACE 2015 software was used to generate the graphs, and all the kinetic trajectories and snapshots were processed through the VMD 1.9.8 visualization software [43].

## 4. Conclusions

In this work, the molecular organization, adsorption, and aggregation behavior of hydrocarbon and pure short-chain fluorocarbon surfactants, as well as their mixed systems, at the gas–liquid interface, were investigated using molecular dynamics simulations. It is observable that by analyzing the equilibrium configurations and concentration distribution curves of PFH_X_A/SDS mixtures. The mixed surfactant molecules accumulate at the interface, with the polar head group of the surfactant oriented towards the aqueous phase and the carbon chain extending into the air and surrounded by counterions bound to it. Consequently, at the air/water interface, the PFH_X_A and SDS surfactants and their mixtures both exhibit good surface activity.

Compared to the pure SDS surfactant system, the carbon chains of the PFH_X_A/SDS hybrid surfactant showed a more stretched conformation and a smaller tilt angle, as demonstrated by molecular dynamics simulations. The mixed surfactants created a highly ordered monolayer at the air/water interface through synergistic interaction. PFH_X_A/SDS surfactants in a mixed structure with a 1:1 molar ratio showed good synergistic interaction at the air/water interface. The calculations of the radial distribution function reveal that the PFH_X_A surfactant affects the interaction of the -OSO_3^−^_ head group with water molecules. The addition of short-chain fluorocarbon surfactants greatly decreased the concentration of Na ions surrounding -OSO_3^−^_ and strengthened the interaction between -OSO_3^−^_ and water molecules, which is helpful in lowering surface tension, as compared to the pure surfactant system. The strongest interaction between the PFH_X_A/SDS mixture surfactant and water molecules occurs when the molar ratio is 1:1, resulting in the best surface properties, according to calculations of the hydrogen bonds between the solute and solvent in the system and the solvent-accessible surface area of the surfactant. The simulation results showed that a mixed PFH_X_A/SDS system with a suitable ratio is better than a pure SDS surfactant system. Our simulation results may provide some insights into the mechanism of synergistic interaction of short-chain fluorocarbon surfactant/hydrocarbon surfactant blend surfactants at the gas/liquid interface.

## Figures and Tables

**Table 1:** Interfacial tension values of systems with different proportional systems of PFH_X_A/SDS.

Surfactant	Surface Tension (mN/m)
n(PFH_X_A)/n(SDS) = 0/4	37.4
n(PFH_X_A)/n(SDS) = 4/0	32.9
n(PFH_X_A)/n(SDS) = 1/3	28.8
n(PFH_X_A)/n(SDS) = 1/1	21.4
n(PFH_X_A)/n(SDS) = 3/1	23.2

**Table 2:** The number of molecules in each PFH_X_A/SDS hybrid system for surfactant simulations at the air–water interface.

Molar Ration of Surfactants in Each Simulate System	Number of Molecules		
	SDS	PFH_X_A	H_2_O
n(PFH_X_A)/n(SDS) = 4/0	0	25	2436
n(PFH_X_A)/n(SDS) = 3/1	6	19	2407
n(PFH_X_A)/n(SDS) = 1/1	13	12	2465
n(PFH_X_A)/n(SDS) = 1/3	19	6	2413
n(PFH_X_A)/n(SDS) = 0/4	25	0	2487

## Data Availability

The data presented will be made available on request from the corresponding authors.

## Supplementary Materials

The following supporting information can be downloaded at: https://www.mdpi.com/article/10.3390/molecules29071606/s1. Figure S1. The 2D and 3D molecular structures of PFHXA monomer (a) and SDS monomer (b). Figure S2. Side view (a) and top view (b) of the PFH_X_A surfactant single layer system on the gas-liquid interface. Figure S3. Density distribution profiles of pure PFH_X_A surfactant at gas-liquid interface. Figure S4. Average RMSD for the PFH_X_A surfactant in the mixed monolayers.
