# Supplementary material for: Molecular Dynamics Simulations of the Short-Chain Fluorocarbon Surfactant PFHXA and the Anionic Surfactant SDS at the Air/Water Interface
Source: Molecules. 2024 Apr 3;29(7):1606. doi: 10.3390/molecules29071606 (PMC11013209; doi:10.3390/molecules29071606)
Supplement: Supplementary file 1 [file molecules-29-01606-s001.zip › molecules-2900899-supplementary.pdf]

Supporting information

Molecular Dynamics Simulations of the Short-Chain Fluorocarbon Surfactant PFHxA and the Anionic Surfactant SDS at the Air/Water Interface

Jinqing Jiao [1,*,†], Tao Li [2,†], Guangwen Zhang [1], Jing Xiong [2], Xuqing Lang [1], Xiaolong Quan [2], Yiwei Cheng [1] and Yuechang Wei [2,*]

1 State Key Laboratory of Chemical Safety, SINOPEC Research Institute of Safety Engineering Co., Ltd., Qingdao 266071, China
2 State Key Laboratory of Heavy Oil Processing, China University of Petroleum (Beijing), Beijing 102249, China
* Correspondence: jiaojq.qday@sinopec.com (J.J.); weiyc@cup.edu.cn (Y.W.)
† These authors contributed equally to this work.

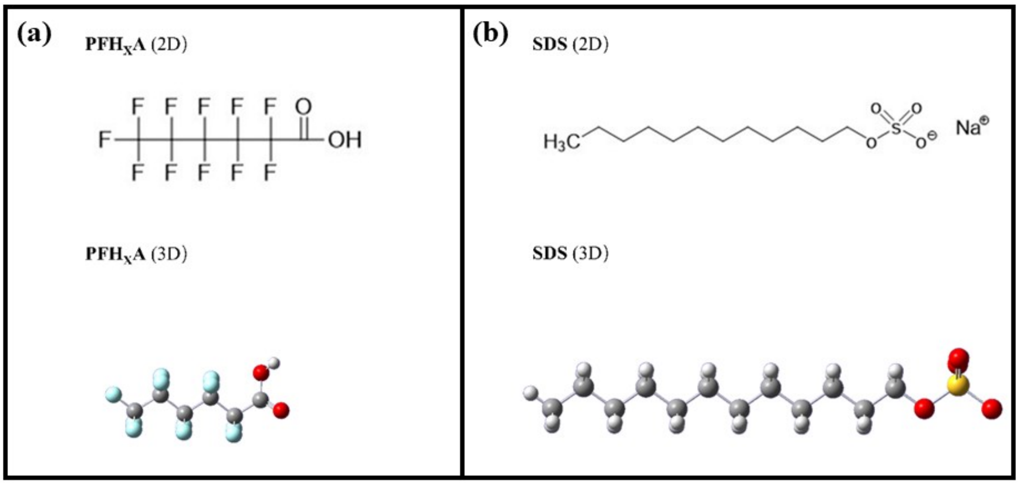

Figure S1. The 2D and 3D molecular structures of PFHXA monomer (a) and SDS monomer (b). The atomic coloring scheme is red (O atoms), grey (C atoms), yellow (S atoms), white (H atoms), blue (F atoms).

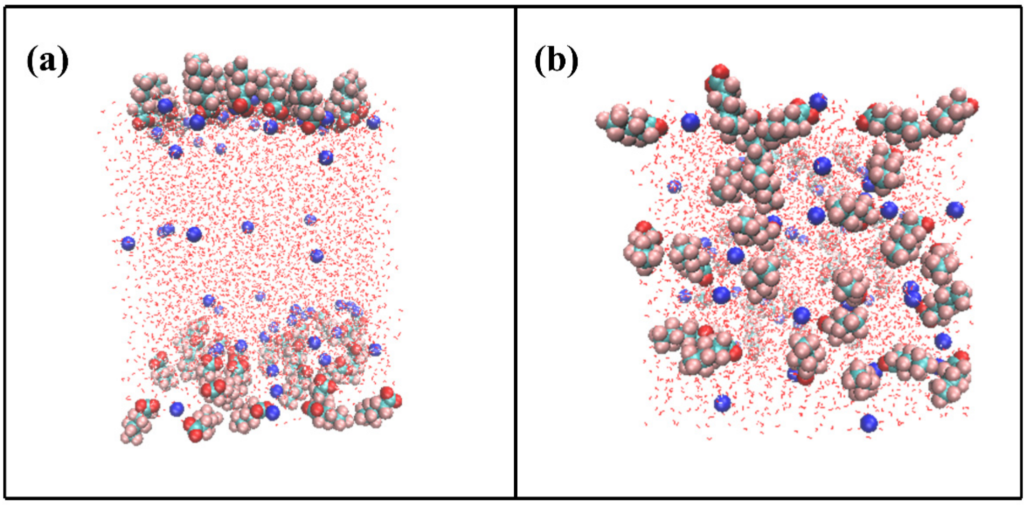

Figure S2. Side view (a) and top view (b) of the PFHxA surfactant single layer system on the gas-liquid interface..

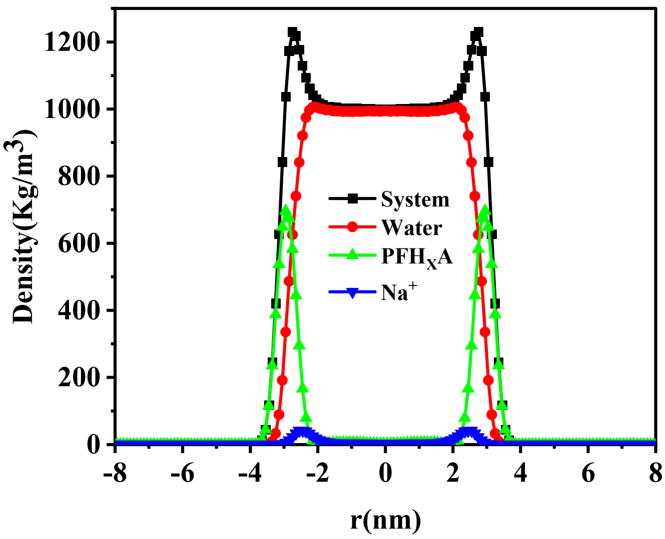

Figure S3. Density distribution profiles of pure PFHxA surfactant at gas-liquid interface.

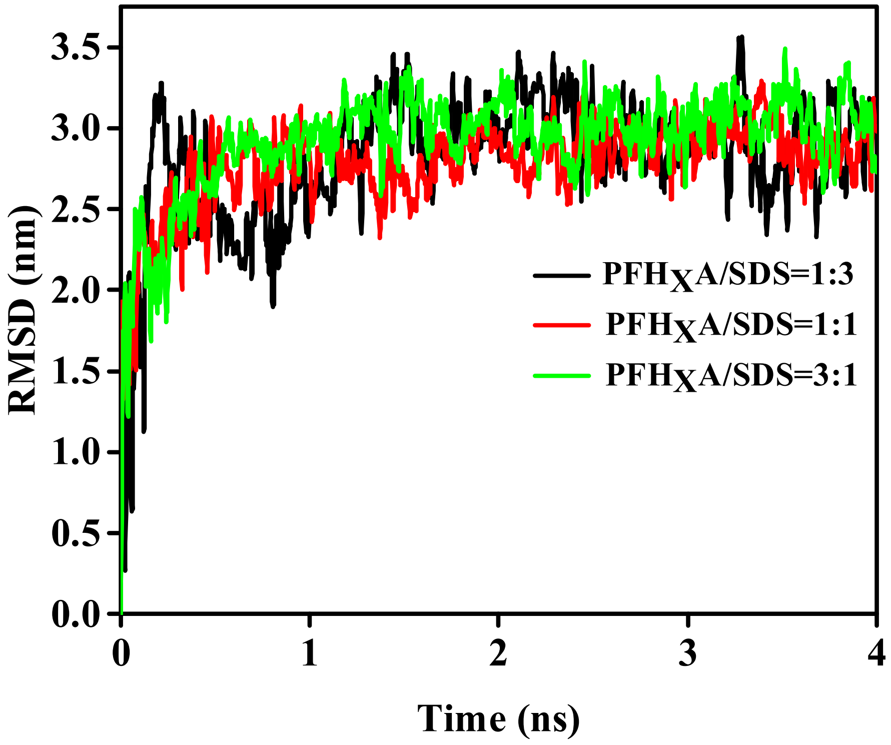

Figure S4. Average RMSD for the PFHxA surfactant in the mixed monolayers.
